# Gold-Catalyzed Complementary Nitroalkyne Internal Redox Process: A DFT Study

**DOI:** 10.3389/fchem.2021.689780

**Published:** 2021-07-09

**Authors:** K. Vipin Raj, Pawan S. Dhote, Kumar Vanka, Chepuri V. Ramana

**Affiliations:** ^1^ Physical and Materials Chemistry Division, CSIR-National Chemical Laboratory, Pune, India; ^2^ Academy of Scientific and Innovative Research (AcSIR), Ghaziabad, India; ^3^ Organic Chemistry Division, CSIR-National Chemical Laboratory, Pune, India

**Keywords:** gold-catalysis, internal redox, cycloisomerization, α-oxo gold carbene, DFT calculation

## Abstract

Gold-catalysis, in this century, is one of the most emerging and promising new areas of research in organic synthesis. During the last two decades, a wide range of distinct synthetic methodologies have been unveiled employing homogeneous gold catalysis and aptly applied in the synthesis of numerous natural products and biologically active molecules. Among these, the reactions involving α-oxo gold carbene/α-imino gold carbene intermediates are of contemporary interest, in view of their synthetic potential and also due to the need to understand the bonding involved in these complexes. In this manuscript, we document the theoretical investigations on the regio-selectivity dependence of substitution on the gold-catalyzed cycloisomerization of *o*-nitroarylalkyne derivatives. We have also studied the relative stabilities of α-oxo gold carbene intermediates.

## Introduction

Gold catalyzed reactions have been increasingly emerging in the literature over the past few decades (Hashmi, 2007; Pflästerera and Hashmi, 2015). The majority of these reactions are based on the propensity of gold complexes to act as carbophilic Lewis acids in the activation of carbon–carbon multiple bonds, thus allowing the formation of new C–C and C–hetero atom bonds by inter-/intramolecular addition of nucleophiles across the Au-complexed multiple bonds (Corma et al., 2011). An interesting class of gold-catalyzed reactions that needs a mention in this context are the catalytic internal redox cyclisation’s (Xiao and Li, 2011; Zhang, 2014; Yeom and Shin, 2014). The oxygen atom transfer to alkynes catalyzed by gold complexes is a well-known addition−elimination process employing nucleophilic oxygen atom donors such as nitro (Asao et al., 2003; Li et al., 2005; Ramana et al., 2010), amine-/pyridine *N*-oxides (Cui et al., 2009; Nosel et al., 2013), nitrone (Heom et al., 2008; Pati and Liu, 2009; Chen et al., 2011), sulfoxides (Shapiro and Toste, 2007; Lu et al., 2013), and epoxides (Hashmi et al., 2008; Lin et al., 2008), reacting with the activated alkynes.

**SCHEME 1:**
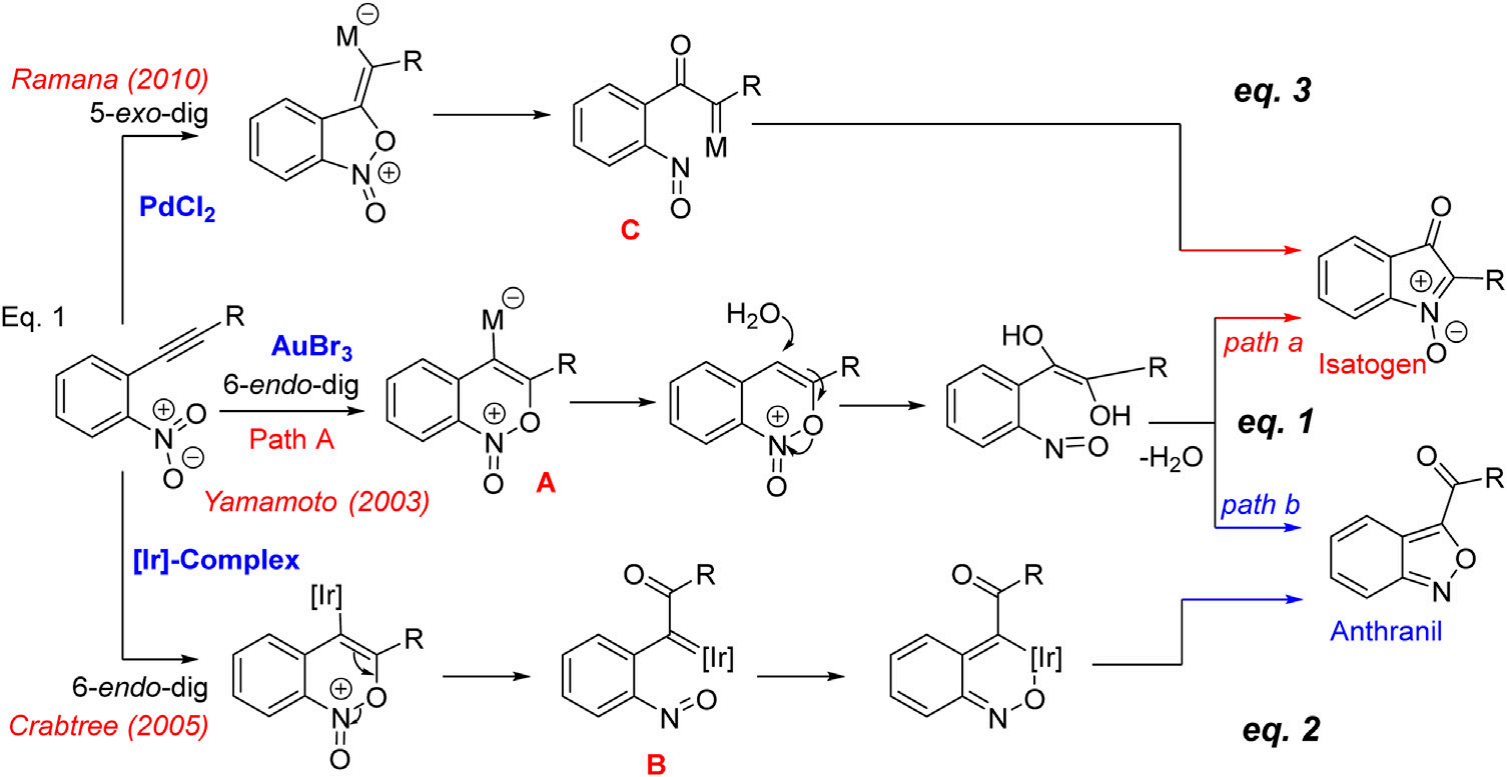
Mechanism of nitroalkyne cycloisomerization.

The Au-catalyzed cycloisomerizaton of nitrotolans documented by Yamamoto and co-workers in 2003 (Scheme 1, Eq. 1) (Asao et al., 2003), is an important advancement to synthesize 2-arylisatogens. Interestingly, when the pendant alkyne substituent is an alkyl group, the internal redox process proceeds in a complementary mode resulting in the formation of a benzo[*c*]isoxazole, trivially known as anthranil. A mechanism founded upon the addition of the oxygen of the nitro group in a 6-*endo*-dig fashion has been postulated as the key step involved for the intramolecular redox process. Initially, it has been proposed that the resulting gold-ate complex **A** undergoes protonolysis followed by ring opening with water to produce a nitrosobenzene. There exist two possibilities for the subsequent dehydrative cyclization leading to isatogens (*path a*) or anthranil (*path b*). Though this explains the possible paths, it does not account for why these paths are substituent dependent. In a later report, Crabtree’s group has documented (Scheme 1, Eq. 2) a similar nitroalkynes cycloisomerization by iridium hydrides leading to anthranils (Li et al., 2005). With the help of single crystal structural analysis, it has been proved that there exists an intermediate iridium(III) nitroso complex **B**, which results after the initial oxygen transfer from nitro to alkyne in a 6-*endo*-dig fashion. In this context, as a part of the total synthesis of the pseudoindoxyl class of natural products (Ramana et al., 2010; Patel et al., 2013), we have speculated on the possibility of complementing this process by employing Pd-complexes which was successfully realized to come up with a general method for the synthesis of 2-aryl and 2-alkyl isatogens *via* an internal nitro-alkyne redox process (Scheme 1, Eq. 3) (Ramana et al., 2010). We have also studied in detail the mechanistic aspects with density functional theory (DFT) and reasoned that the formation of an α-oxo metal carbenoid **C** occurs by the 5-*exo*-dig mode of cyclization of the nitro group on the alkyne, which subsequently undergoes a 6n-electro cyclization to isatogen. In this manuscript, we document the DFT calculations on the [Au]-catalysed complementary nitroalkyne redox process that leads to α-oxo gold carbenes **B** and **C**, especially focusing on the energies associated with oxygen transfer and carbene transfer. This has been undertaken considering the importance of gold catalyzed processes that proceed through the α-imino and/or α-oxo gold carbenes and their promising applications in the heterocyclic synthesis (Aguilar and Santamaría, 2019; Zhang, 2014). These processes in general proceed through the carbene and/or nitrene transfer from the [Au]-centre (Ye, 2020). However, in case of the Au-carbenes **B** and **C**, such a transfer is challenging, as the internal electrocyclization along with the nitroso group is highly favored with either of them. The possibility of trapping these reactive intermediates has been attempted with internal and external nucleophiles. However, on both instances, it has been realized that the intramolecular process leading to isatogens or anthranil has exclusively taken place and the products obtained are from the reaction of the nucleophile employed with the isatogen. Thus, a qualitative understanding of the relative energies of **B** and **C** and the energies associated in their formation is expected to provide some clues on the possible trapping.

## Results and Discussion

**SCHEME 2:**
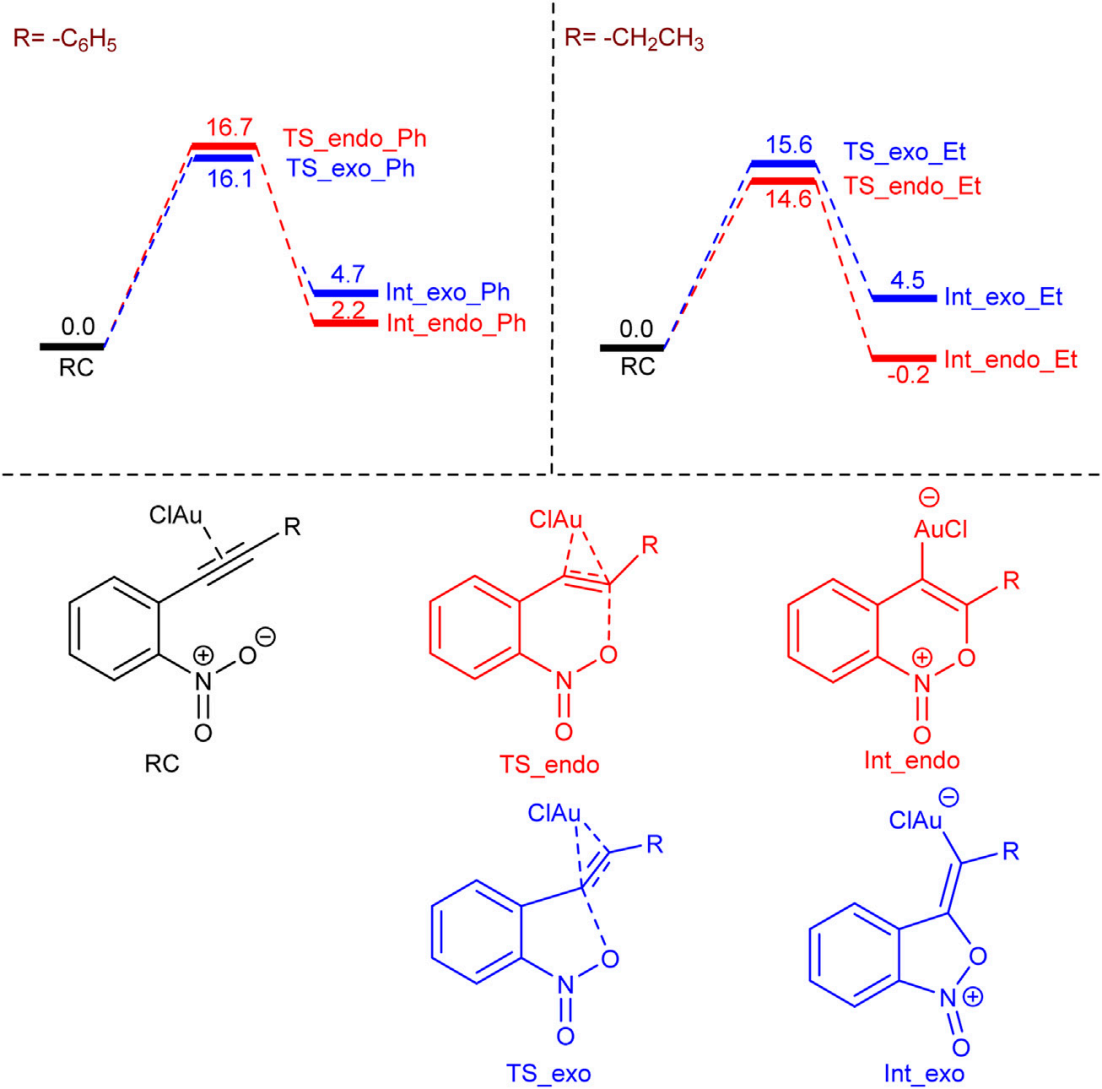
The free energy profile for the formation of -*endo* and -*exo* intermediates for –phenyl and –ethyl substitutions. Values are in kcal/mol.

In order to understand the selectivity (*endo* or *exo*) dependence on the substitution (alkyl or aromatic) on the nitro alkyne, we have performed density functional theory (DFT) calculations using the Turbomole 7.2 program package (TURBOMOLE V7.2, 2017). We have chosen the ethyl (–CH_2_CH_3_) and phenyl (–C_6_H_5_) groups as the representatives for alkyl and aromatic substitutions and considered AuCl as the catalyst instead of AuCl_3_, in accordance with previously reported work (Straub, 2004). The *endo* or *exo* selectivity arises due to the two different possibilities of oxygen (of the nitro group) attack on the C–C triple bond (see Scheme 2), and our calculations indicate that the *exo* transition state (TS_exo_Ph) is favorable by 0.6 kcal/mol over the *endo* transition state (TS_endo_Ph) for phenyl substitution (see Scheme 2). In contrast, the *endo* transition state (TS_endo_Et) is favorable by 1.0 kcal/mol over the *exo* transition (TS_exo_Et) state for ethyl substitution, which corroborates with the experimental observations. Furthermore, in the case of ethyl substitution, the *endo* intermediate (Int_endo_Et) is more preferable than the *exo* intermediate (Int_exo_Et) by 4.7 kcal/mol, but in the case of phenyl substitution, the *exo* intermediate (Int_exo_Ph) is less favorable than the *endo* intermediate (Int_endo_Ph) by 2.5 kcal/mol. In other words, the *endo* pathway to form the first intermediate is favorable both kinetically and thermodynamically for the ethyl substitution, but the *exo* pathway is only kinetically favorable for the phenyl substitution.

**FIGURE 1:**
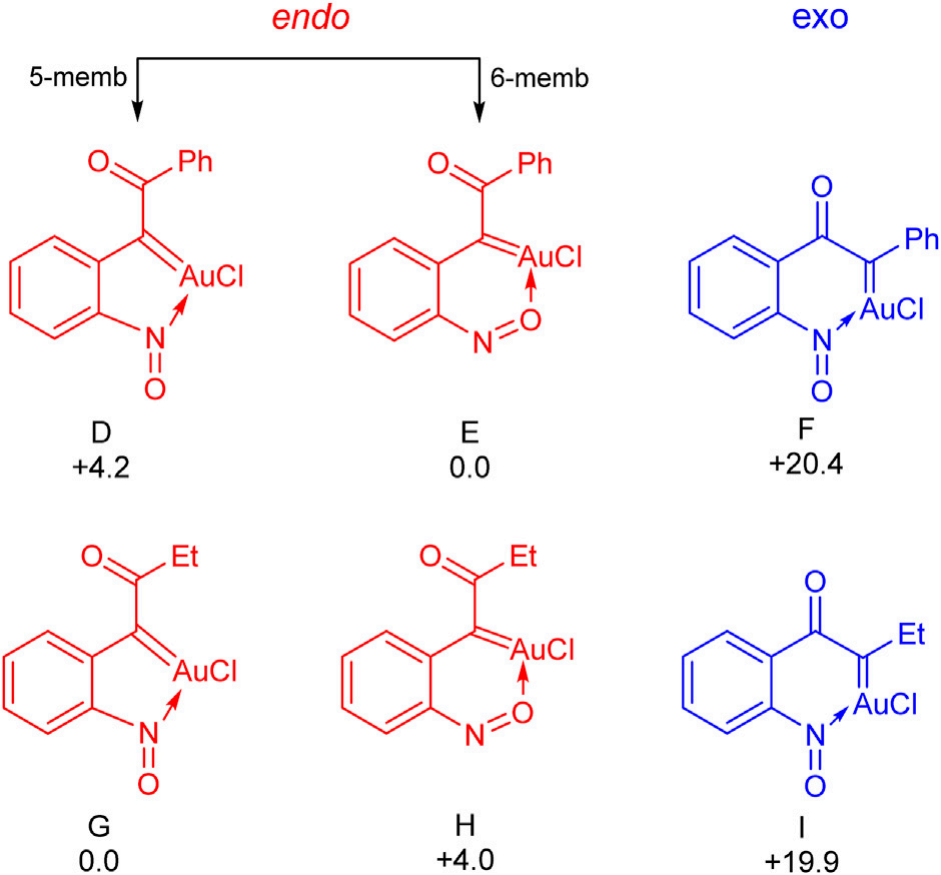
The relative free energies of α-oxo gold carbenes with respect to the most stable one (E for the phenyl substitution and G for the ethyl substitution). Values are in kcal/mol.

Next, the energies of the six different possible oxo gold carbenes (D to I in Figure 1) have been calculated to see their relative stability. As shown in Figure 1, depending upon the heteroatom of the nitroso group involved in the coordination, there exists two possibilities for the oxo gold carbene resulting from the 6-*endo* dig path – with the nitrogen atom, a five–membered coordination (D and G for Ph and Et respectively) and six-membered coordination with the oxygen atom (E and H for Ph and Et respectively). Interestingly, with both phenyl and ethyl, the [Au]-carbenes derived from the 6-*endo* dig path are more stable. In case of the phenyl, E, the six-membered coordination is more stable than the D, five-membered coordination, by 4.2 kcal/mol. However, with the ethyl substituent, this was seen to be exactly reversed. G, the five-membered coordination was seen to be more stable than H, the six-membered coordination, by 4.0 kcal/mol. When it comes to the 5-*exo* dig path, in both the ethyl (19.9 kcal/mol unfavourable compared to G) and phenyl (20.4 kcal/mol unfavourable compared to E) cases, the energies associated with resulting carbenes reveal that they are unfavourable.

Overall, these preliminary calculations indicate that the α-oxo gold carbenes resulting from the 6-*endo*-dig mode of oxygen transfer are favored in general, though the energies associated with this process vary with respect to the substituent. For example, as discussed already, the formation of the first intermediate *via* the 6-*endo*-dig mode is kinetically and thermodynamically favourable for ethyl substitution. However, the corresponding mode is only thermodynamically favourable for phenyl substitution. This indicates that energies associated with the internal oxygen transfer lead to the α-oxo gold carbene B and also, given its comparable stability, that the chances of the carbene transfer from this reactive intermediate are greater.

## Conclusion

To conclude, DFT calculations on the internal oxygen transfer of the Au-catalyzed *o*-nitroalkyne cycloisomerization reactions have been carried out to understand the relative energies associated with the oxygen transfer and the energies of the resulting α-oxo gold carbenes. These calculations clearly reveal that the α-oxo gold carbenes resulting from the 6-*endo* dig addition of the oxygen to the alkyne is thermodynamically stable, when compared to the alternative α-oxo gold carbene that results from the 5-*exo* dig addition. Our calculations also suggest that the substitutions on the *o*-nitroalkynes have a significant role in altering the regio-selectivity of the reaction.

## Computational Details

All the calculations in this study have been performed with density functional theory (DFT), with the aid of the Turbomole 7.2 suite of programs (TURBOMOLE V7.2, 2017), using the M06-2X functional (Zhao and Truhlar, 2008). The def-TZVP basis set has been employed (Schäfer et al., 1994; Eichkorn et al., 1997). The resolution of identity (RI) (Eichkorn et al., 1995), along with the multipole accelerated resolution of identity (marij) (Sierka et al., 2003) approximations have been employed for an accurate and efficient treatment of the electronic Coulomb term in the DFT calculations. Solvent correction were incorporated with optimization calculations using the COSMO model (Klamt and Schüürmann, 1993), with toluene (*ε* = 2.374) as the solvent. The values reported are ΔG values, with zero-point energy corrections, internal energy and entropic contributions were included through frequency calculations on the optimized minima, with the temperature taken to be 298.15 K. Harmonic frequency calculations were performed for all stationary points to confirm them as a local minima or transition state structures. The XYZ coordinates of the optimized geometries of all the structures are provided in the Supplementary Material.

## Data Availability

The original contributions presented in the study are included in the article/Supplementary Material, further inquiries can be directed to the corresponding author.

## References

[B1] Aguilar E. Santamaría J. (2019). Gold-catalyzed Heterocyclic Syntheses through α-imino Gold Carbene Complexes as Intermediates. Org. Chem. Front. 6, 1513–1540. 10.1039/C9QO00243J

[B2] Asao N. Sato K. Yamamoto Y. (2003). AuBr3-catalyzed Cyclization of O-(alkynyl)nitrobenzenes. Efficient Synthesis of Isatogens and Anthranils. Tetrahedron Lett. 44, 5675–5677. 10.1016/S0040-4039(03)01357-1

[B3] Chen D. Song G. Y. Jia A. Q. Li X. W. (2011). Gold- and Iodine-Mediated Internal Oxygen Transfer of Nitrone- and Sulfoxide-Functionalized Alkynes. J. Org. Chem. 76, 8488–8494. 10.1021/jo201347r 21902170

[B4] Corma A. Leyva-Pérez A. Sabater M. J. (2011). Gold-Catalyzed Carbon−Heteroatom Bond-Forming Reactions. Chem. Rev. 111 (3), 1657–1712. 10.1021/cr100414u 21391565

[B5] Cui L. Peng Y. Zhang L. (2009). A Two-step, Formal [4 + 2] Approach toward Piperidin-4-Ones via Au Catalysis. J. Am. Chem. Soc. 131, 8394–8395. 10.1021/ja903531g 19492799

[B6] Eichkorn K. Treutler O. Ӧhm H. Häser M. Ahlrichs R. Ӧhm H. (1995). Auxiliary Basis Sets to Approximate Coulomb Potentials. Chem. Phys. Lett. 240, 283–290. 10.1016/0009-2614(95)00621-A

[B7] Eichkorn K. Weigend F. Treutler O. Ahlrichs R. (1997). Auxiliary Basis Sets for Main Row Atoms and Transition Metals and Their Use to Approximate Coulomb Potentials. Theor. Chem. Acc. Theor. Comput. Model. (Theoretica Chim. Acta). 97, 119–124. 10.1007/s002140050244

[B8] Hashmi A. S. K. Bührle M. Salathé R. Bats J. W. (2008). Gold Catalysis: Synthesis of 3-Acylindenes from 2-Alkynylaryl Epoxides. *Adv. Synth* . Adv. Synth. Catal. 350, 2059–2064. 10.1002/adsc.200800385

[B9] Hashmi A. S. K. (2007). Gold-Catalyzed Organic Reactions. Chem. Rev. 107, 3180–3211. 10.1021/cr000436x 17580975

[B10] Heom H. S. Lee J. E. Shin S. (2008). Gold-Catalyzed Waste-free Generation and Reaction of Azomethine Ylides: Internal Redox/Dipolar Cycloaddition Cascade. Angew. Chem. Int. Ed. 47, 7040–7043. 10.1002/anie.200802802 18677734

[B11] Klamt A. Schüürmann G. (1993). COSMO: a New Approach to Dielectric Screening in Solvents with Explicit Expressions for the Screening Energy and its Gradient. J. Chem. Soc. Perkin Trans. 2, 799–805. 10.1039/P29930000799

[B12] Li X. Incarvito C. D. Vogel T. Crabtree R. H. (2005). Intramolecular Oxygen Transfer from Nitro Groups to C⋮C Bonds Mediated by Iridium Hydrides. Organometallics. 24, 3066–3073. 10.1021/om050116+

[B13] Lin G.-Y. Li C.-W. Hung S.-H. Liu R.-S. (2008). Diversity in Gold- and Silver-Catalyzed Cycloisomerization of Epoxide−Alkyne Functionalities. Org. Lett. 10, 5059–5062. 10.1021/ol802047g 18855406

[B14] Lu B. Li Y. Wang Y. Aue D. H. Luo Y. Zhang L. (2013). [3,3]-Sigmatropic Rearrangement versus Carbene Formation in Gold-Catalyzed Transformations of Alkynyl Aryl Sulfoxides: Mechanistic Studies and Expanded Reaction Scope. J. Am. Chem. Soc. 135, 8512–8524. 10.1021/ja401343p 23731178 PMC3704346

[B15] Nösel P. dos Santos Comprido L. N. T. Lauterbach M. Rominger F. Hashmi A. S. K. (2013). 1,6-Carbene Transfer: Gold-Catalyzed Oxidative Diyne Cyclizations. J. Am. Chem. Soc. 135, 15662–15666. 10.1021/ja4085385 24050384

[B16] Patel P. Reddy B. N. P. Ramana C. V. (2013). The Synthesis of the central Tricyclic Core of the Isatisine A: Harmonious Orchestration of [metal]-Catalyzed Reactions in a Sequence. Tetrahedron. 70, 510–516. 10.1016/j.tet.2013.11.026

[B17] Pati K. Liu R. S. (2009). Efficient Syntheses of α-pyridones and 3(2H)-Isoquinolones through Ruthenium-Catalyzed Cycloisomerization of 3-En-5-Ynyl and O-Alkynylphenyl Nitrones Chem. Commun.14, 5233–5235. 10.1039/B910773H 19707630

[B18] Pflästerera D. Hashmi S. (2015). Gold Catalysis in Total Synthesis – Recent Achievements. Chem. Soc. Rev. 45, 1331–1367. 10.1039/C5CS00721F 26673389

[B19] Ramana C. V. Patel P. Vanka K. Miao B. C. Degterev A. (2010). A Combined Experimental and Density Functional Theory Study on the Pd-Mediated Cycloisomerization of O-Alkynylnitrobenzenes—Synthesis of Isatogens and Their Evaluation as Modulators of ROS-Mediated Cell Death. Eur. J. Org. Chem. 2010 (31):5955–5966. 10.1002/ejoc.201000769

[B20] Schäfer A. Huber C. Ahlrichs R. (1994). Fully Optimized Contracted Gaussian Basis Sets of Triple Zeta Valence Quality for Atoms Li to Kr. J. Chem. Phys. 100, 5829–5835. 10.1063/1.467146

[B21] Shapiro N. D. Toste F. D. (2007). Rearrangement of Alkynyl Sulfoxides Catalyzed by Gold(I) Complexes. J. Am. Chem. Soc. 129, 4160–4161. 10.1021/ja070789e 17371031

[B22] Sierka M. Hogekamp A. Ahlrichs R. (2003). Fast Evaluation of the Coulomb Potential for Electron Densities Using Multipole Accelerated Resolution of Identity Approximation. J. Chem. Phys. 118, 9136–9148. 10.1063/1.1567253

[B23] Straub B. F. (2004). Gold(i) or Gold(iii) as Active Species in AuCl3-Catalyzed Cyclization/cycloaddition Reactions? Chem. Commun. 2004, 1726–1728. 10.1039/B404876H 15278157

[B24] TURBOMOLE V7.2 (2017). A development of University of Karlsruhe and Forschungszentrum Karlsruhe GmbH, 1989-2007, TURBOMOLE GmbH, since 2007. Available at: http://www.turbomole.com (Accessed May 18, 2018)

[B25] Xiao J. Li X. (2011). Gold α-Oxo Carbenoids in Catalysis: Catalytic Oxygen-Atom Transfer to Alkynes, Angew. Chem. Int. Ed. 50, 7226–7236. 10.1002/anie.201100148 21726021

[B26] Ye L. W. Zhu X. Sahani R. Xu Y. Qian P. C. Liu R. S. (2020). Nitrene Transfer and Carbene Transfer in Gold Catalysis. Chem. Rev. 2020, 121. 10.1021/acs.chemrev.0c00348 32786423

[B27] Yeom H.-S. Shin S. (2014). Catalytic Access to α-Oxo Gold Carbenes by N-O Bond Oxidants. Acc. Chem. Res. 47, 966–977. 10.1021/ar4001839 24517590

[B28] Zhang L. (2014). A Non-diazo Approach to α-Oxo Gold Carbenes via Gold-Catalyzed Alkyne Oxidation. Acc. Chem. Res. 47, 877–888. 10.1021/ar400181x 24428596 PMC3983127

[B29] Zhao Y. Truhlar D. G. (2008). The M06 Suite of Density Functionals for Main Group Thermochemistry, Thermochemical Kinetics, Noncovalent Interactions, Excited States, and Transition Elements: Two New Functionals and Systematic Testing of Four M06-Class Functionals and 12 Other Functionals. Theor. Chem. Account. 120, 215–241. 10.1007/s00214-007-0310-x

