# Supplementary material for: Gold-Catalyzed Complementary Nitroalkyne Internal Redox Process: A DFT Study
Source: Front Chem. 2021 Jul 9;9:689780. doi: 10.3389/fchem.2021.689780 (PMC8299333; doi:10.3389/fchem.2021.689780)
Supplement: Supplementary file 1 [file datasheet1.docx]

Supplementary Material

# Barriers for the formation of exo and endo transition states of Path A shown in Scheme 1.

**Figure S1**. The barriers for the formation of *exo* and *endo* transition states of Path A shown in Scheme 1. Values are in kcal/mol.

# DOS Plots of the geometries shown in Scheme 2.

The DOS plots given below were generated by using the Multiwfn software package.^S1^ The final geometries from the Turbomole calculations were used to perform single-point calculations at M06-2X functional and 6-31g* basis set for all the atoms except for Au (for Au, we have used LANL2DZ basis set) using the Gaussian 09 software package.^S2^ We have also used the PCM solvent model (toluene as solvent) to get accurate results.

**References**

S1. Tian Lu, Feiwu Chen, Multiwfn: A Multifunctional Wavefunction Analyzer, J. Comput. Chem. 33, 580-592 (2012).

S2. Gaussian 09, Revision A.01, M. J. Frisch, G. W. Trucks, H. B. Schlegel, G. E. Scuseria, M. A. Robb, J. R. Cheeseman, G. Scalmani, V. Barone, G. A. Petersson, H. Nakatsuji, X. Li, M. Caricato, A. Marenich, J. Bloino, B. G. Janesko, R. Gomperts, B. Mennucci, H. P. Hratchian, J. V. Ortiz, A. F. Izmaylov, J. L. Sonnenberg, D. Williams-Young, F. Ding, F. Lipparini, F. Egidi, J. Goings, B. Peng, A. Petrone, T. Henderson, D. Ranasinghe, V. G. Zakrzewski, J. Gao, N. Rega, G. Zheng, W. Liang, M. Hada, M. Ehara, K. Toyota, R. Fukuda, J. Hasegawa, M. Ishida, T. Nakajima, Y. Honda, O. Kitao, H. Nakai, T. Vreven, K. Throssell, J. A. Montgomery, Jr., J. E. Peralta, F. Ogliaro, M. Bearpark, J. J. Heyd, E. Brothers, K. N. Kudin, V. N. Staroverov, T. Keith, R. Kobayashi, J. Normand, K. Raghavachari, A. Rendell, J. C. Burant, S. S. Iyengar, J. Tomasi, M. Cossi, J. M. Millam, M. Klene, C. Adamo, R. Cammi, J. W. Ochterski, R. L. Martin, K. Morokuma, O. Farkas, J. B. Foresman, and D. J. Fox, Gaussian, Inc., Wallingford CT, 2016.


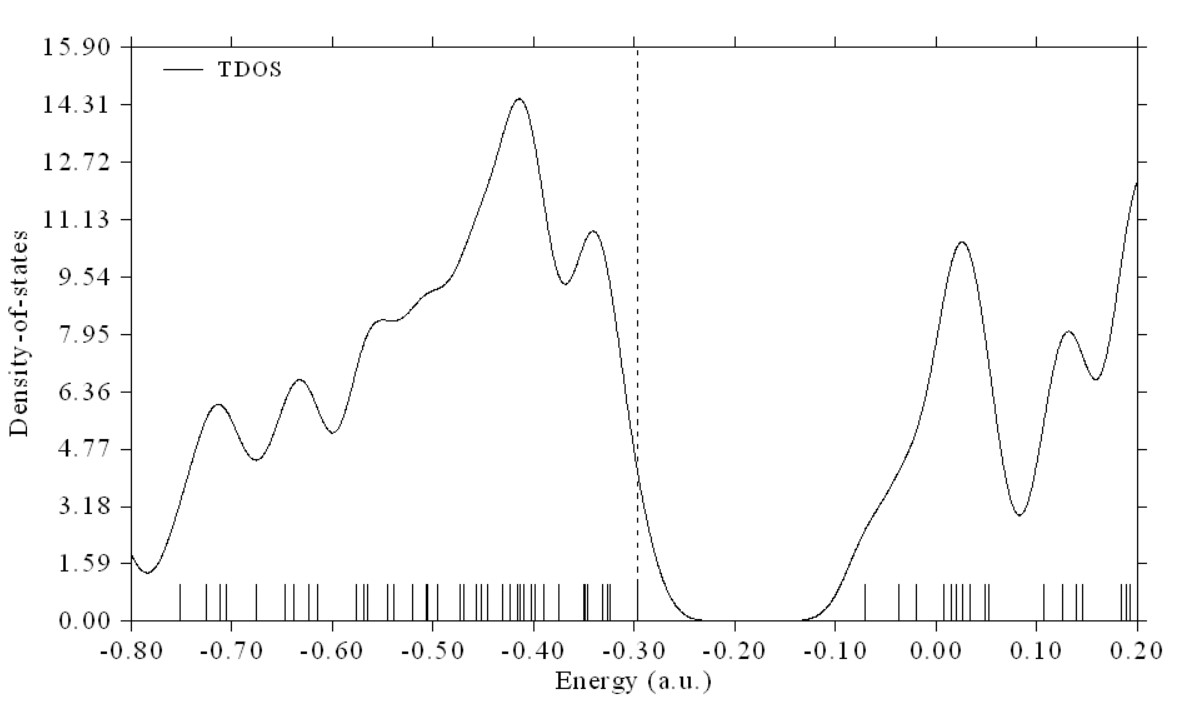


RC (Phenyl)


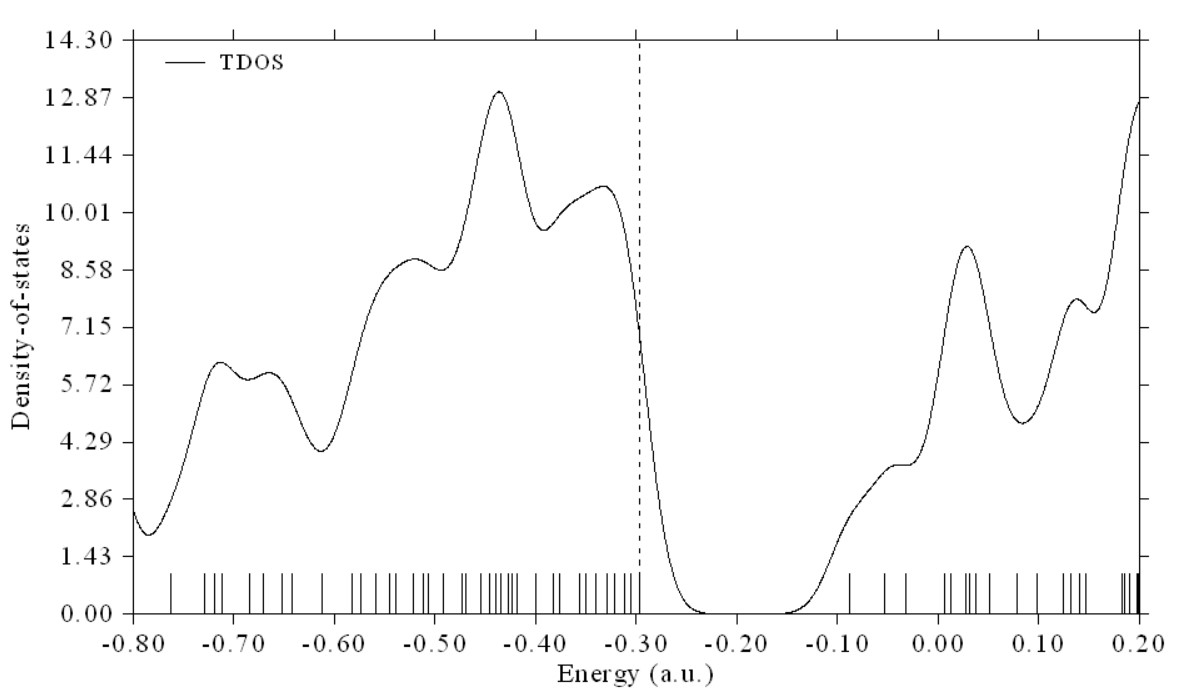


TS_endo_Ph


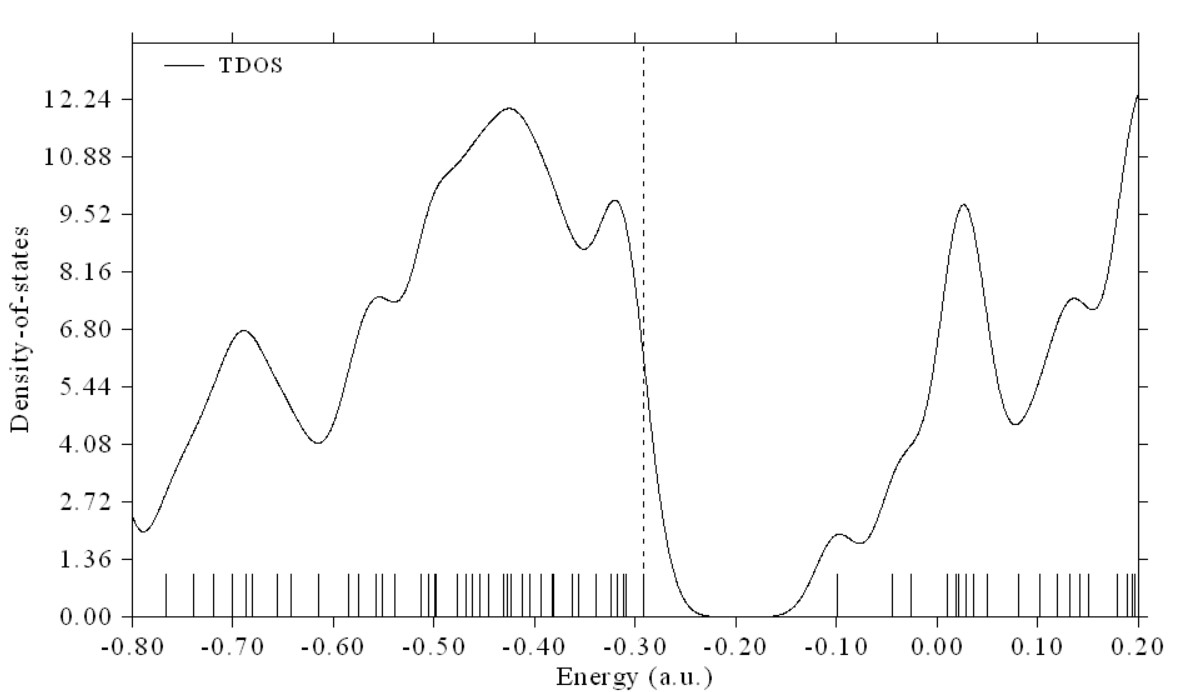


TS_exo_Ph


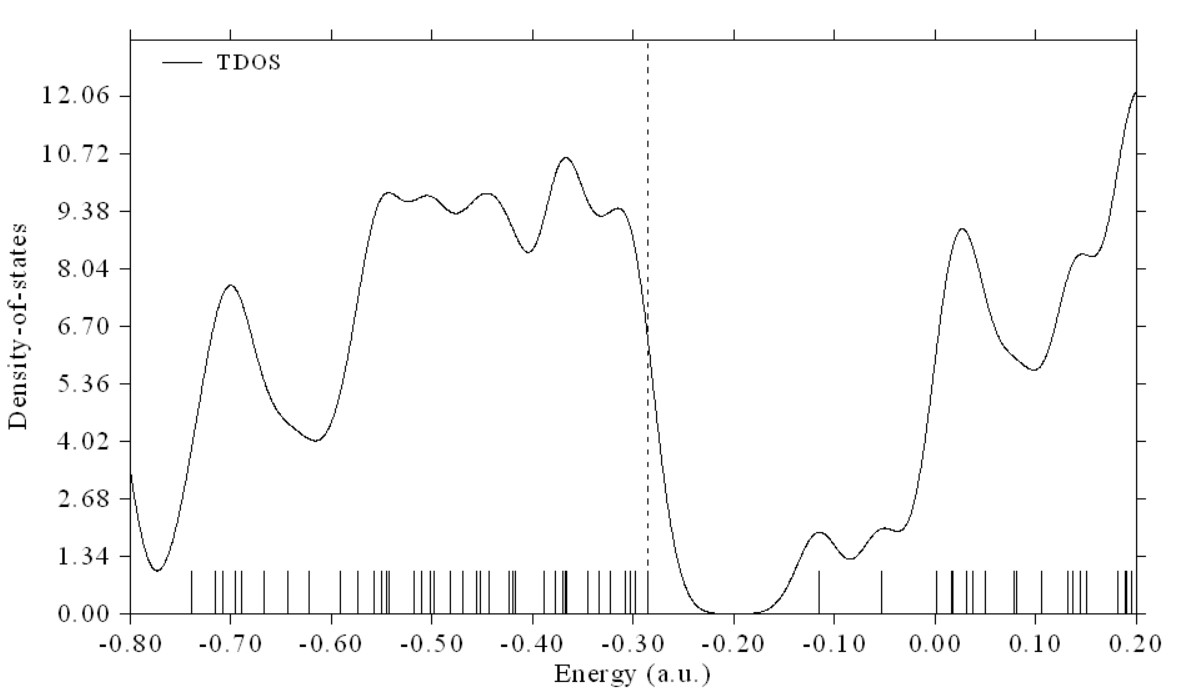


Int_endo_Ph


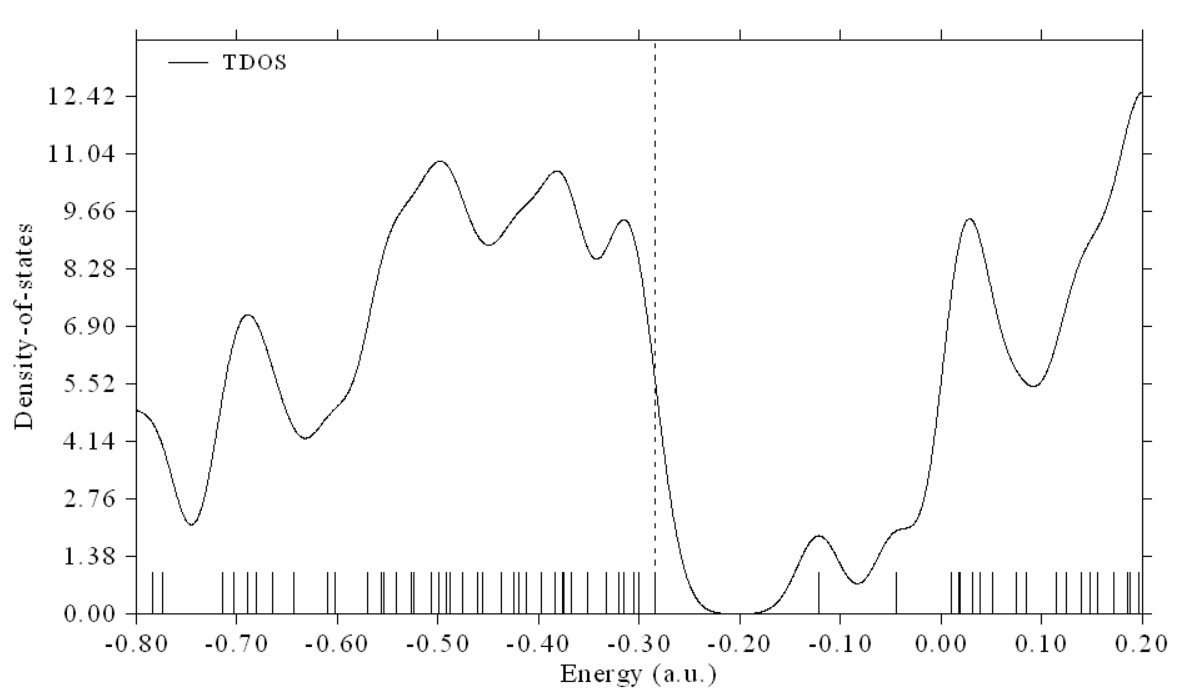


Int_exo_Ph


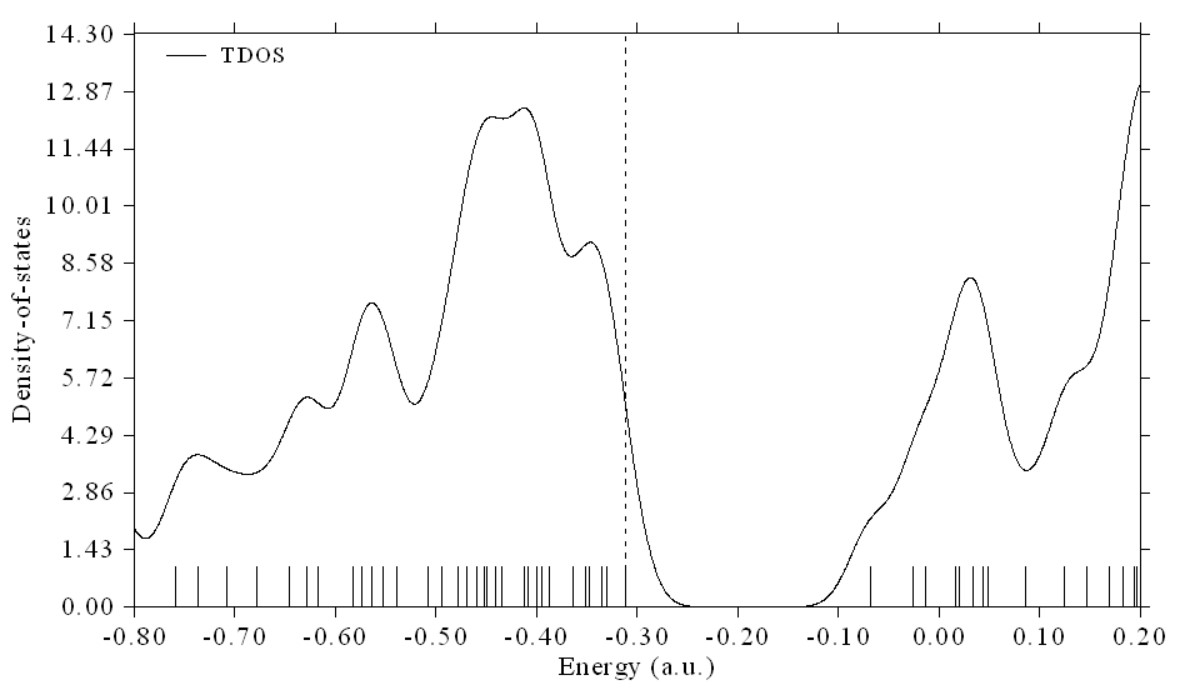


RC (Ethyl)


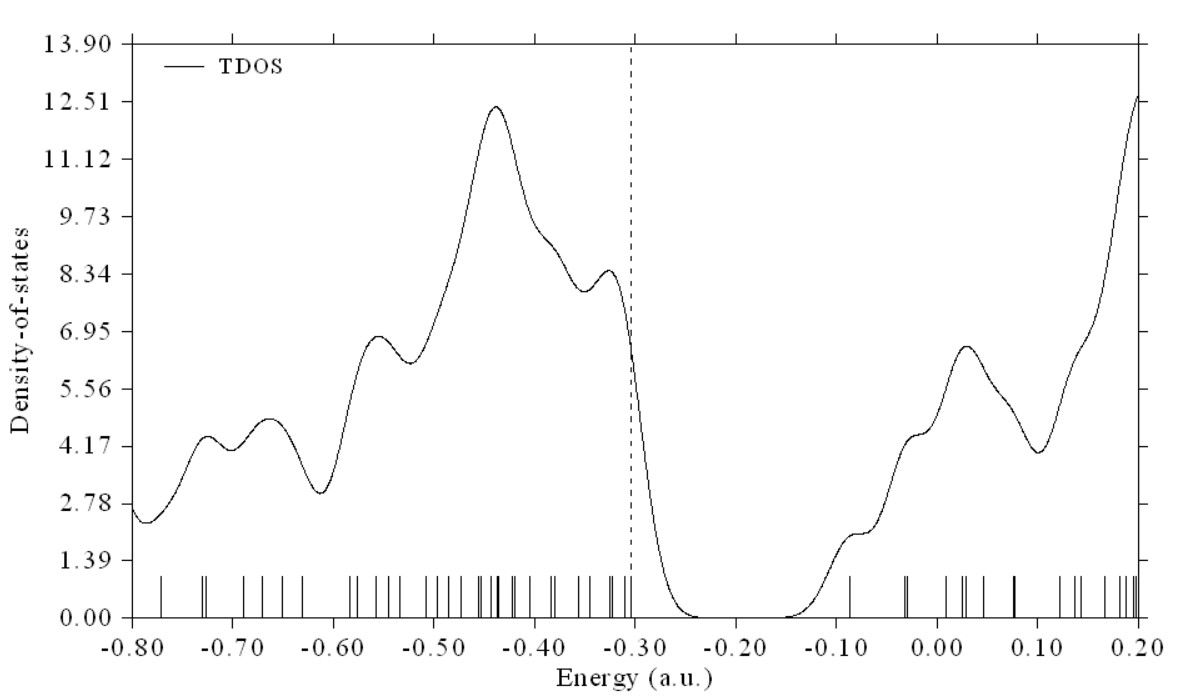


TS_endo_Et


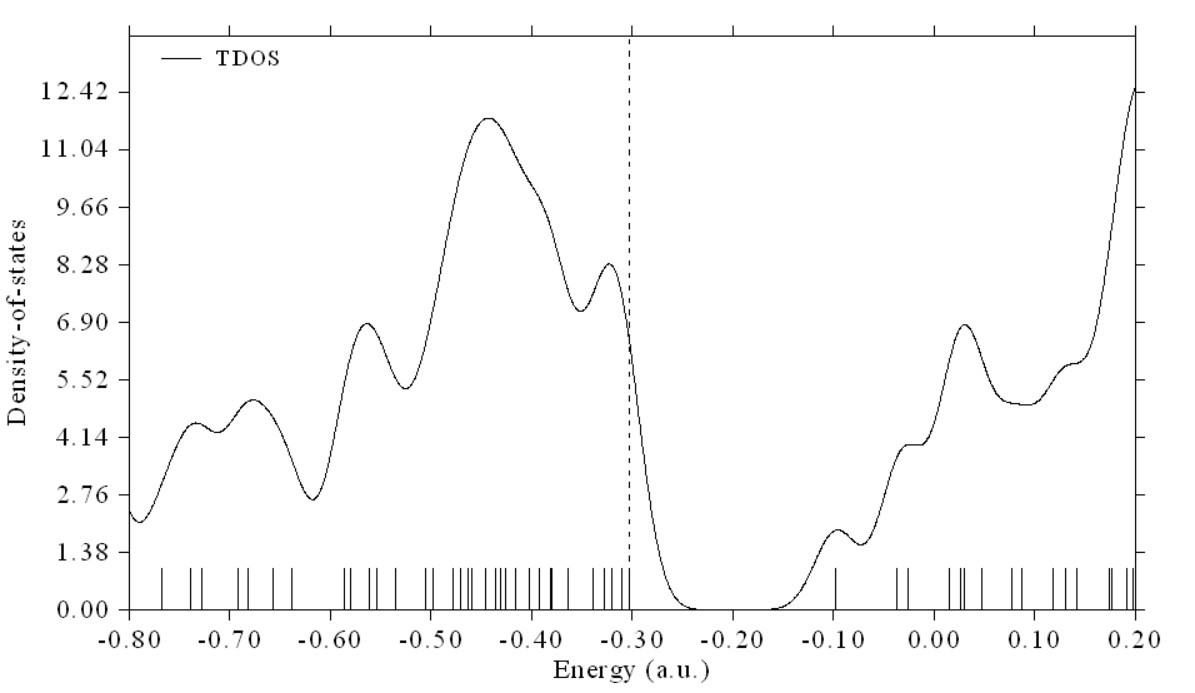


TS_exo_Et


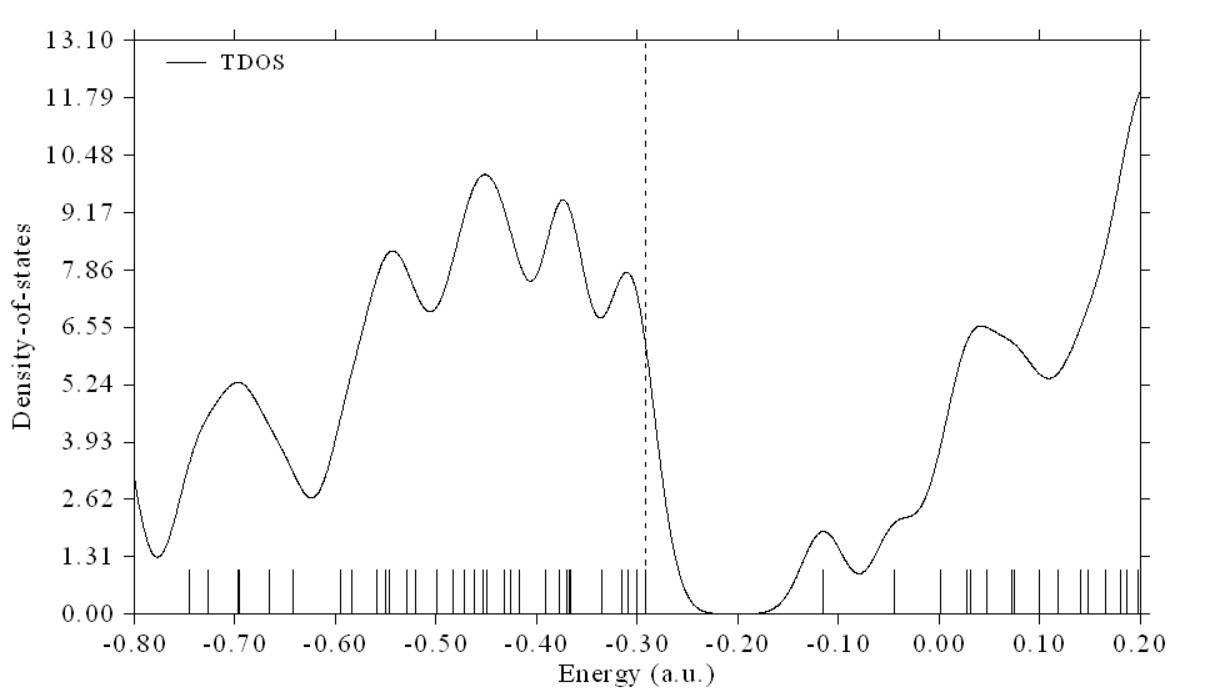


Int_endo_Et


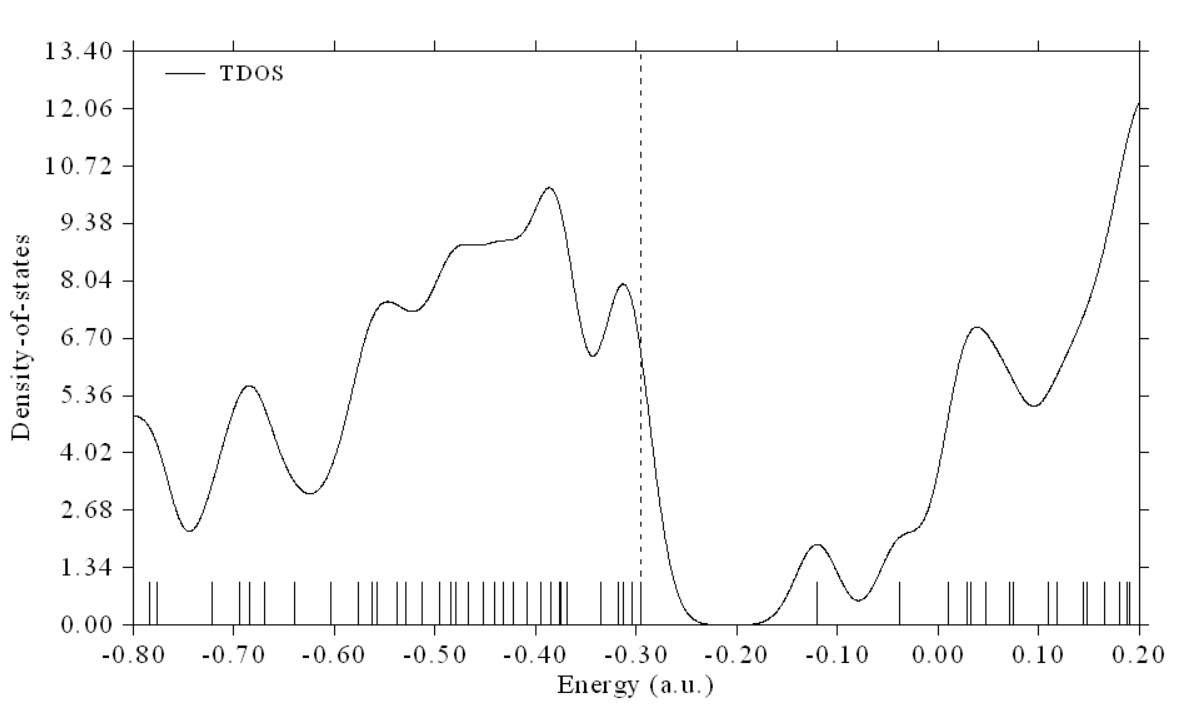


Int_exo_Et

# M06-2X/def-TZVP optimized geometries for the reaction complexes, transition states, intermediates, and various gold carbenes shown in Scheme 2 and Figure 1.

(**1**) RC (R = -C_6_H_5_)

28

C -3.192530 -2.824083 -0.046544

C -2.036688 -2.061151 -0.073559

C -4.438406 -2.219559 0.041318

H -1.074682 -2.547596 -0.150969

H -5.341632 -2.812188 0.070199

C -2.081675 -0.664807 -0.004624

C -4.521014 -0.839299 0.088683

H -5.471296 -0.328921 0.146906

C -3.360772 -0.086160 0.063133

H -3.114940 -3.900928 -0.095583

N -3.540816 1.368581 0.110027

O -2.609056 2.050380 0.465485

O -4.621335 1.808758 -0.204075

C -0.859152 0.102146 -0.026172

C -0.008194 0.997916 -0.001758

Au 1.128762 -0.889029 0.054972

Cl 2.840364 -2.465752 0.166361

C 0.670612 2.267939 -0.011512

C 1.926567 2.433218 0.581237

C 0.035288 3.358199 -0.618633

C 2.534911 3.677645 0.570175

C 0.656839 4.596299 -0.625799

C 1.903452 4.758570 -0.033253

H 2.418028 1.591000 1.051300

H -0.936464 3.226264 -1.072284

H 3.504799 3.802813 1.032421

H 0.167010 5.437361 -1.097999

H 2.384278 5.727656 -0.041998

(**2**) TS_endo_Ph

28

C -4.130817 -0.513061 0.529651

C -2.838924 -0.720167 0.082988

C -4.527384 0.730247 1.018366

H -2.537826 -1.684284 -0.303518

H -5.536648 0.887599 1.371198

C -1.886832 0.302868 0.119393

C -3.623926 1.771066 1.047954

H -3.898913 2.750013 1.411469

C -2.322754 1.542075 0.610006

H -4.839960 -1.328983 0.497164

N -1.431453 2.676702 0.654519

O -0.209621 2.510825 0.522912

O -1.882146 3.772577 0.836594

C -0.527272 0.075731 -0.345911

C 0.473020 0.859704 -0.234623

Au 0.084124 -1.629217 -1.384064

Cl 0.647076 -3.582079 -2.582441

C 1.863315 1.216925 -0.296195

C 2.808851 0.355563 0.274973

C 2.278442 2.394655 -0.929501

C 4.155235 0.672018 0.203744

C 3.626031 2.706807 -0.980809

C 4.562648 1.848963 -0.413978

H 2.479663 -0.556226 0.755761

H 1.542867 3.053967 -1.368698

H 4.887176 0.003830 0.636686

H 3.949714 3.617333 -1.467141

H 5.614954 2.098791 -0.454926

(**3**) TS_exo_Ph

28

C -4.041742 -0.674116 0.135152

C -2.677502 -0.803550 -0.068080

C -4.650925 0.563576 0.369420

H -2.241341 -1.775922 -0.248862

H -5.718558 0.622633 0.524014

C -1.875987 0.343269 -0.034299

C -3.884816 1.710034 0.406103

H -4.300502 2.691933 0.586102

C -2.525021 1.552155 0.201099

H -4.654429 -1.564736 0.110793

N -1.613591 2.645594 0.236198

O -0.426142 2.319071 0.051066

O -1.958702 3.772335 0.433546

C -0.449708 0.438363 -0.210086

C 0.709983 -0.056210 -0.423234

Au 0.365967 -2.117529 -0.656410

Cl 0.024591 -4.432500 -0.953071

C 2.063483 0.474295 -0.568333

C 3.169913 -0.297038 -0.193665

C 2.272804 1.749849 -1.105890

C 4.453287 0.210061 -0.324388

C 3.559660 2.240524 -1.254225

C 4.651268 1.477061 -0.858653

H 3.018817 -1.292626 0.204111

H 1.427401 2.343198 -1.421137

H 5.301343 -0.390567 -0.023617

H 3.710753 3.222411 -1.682437

H 5.654358 1.865469 -0.976667

(**4**) Int_endo_Ph

28

C -3.207350 2.733135 0.354044

C -2.350983 1.666980 0.378703

C -2.742536 4.059380 0.165112

H -2.715655 0.658497 0.516569

H -3.444739 4.881443 0.149320

C -0.955163 1.850587 0.223521

C -1.413937 4.298494 0.001157

H -1.011406 5.289503 -0.148547

C -0.538071 3.189118 0.037347

H -4.268384 2.564847 0.478328

N 0.796680 3.442050 -0.124778

O 1.669756 2.450566 -0.052762

O 1.282565 4.524750 -0.314374

C -0.019173 0.770227 0.233290

C 1.289323 1.135603 0.108207

Au -0.614716 -1.168736 0.307649

Cl -1.336829 -3.436290 0.343793

C 2.505232 0.304547 0.104143

C 2.614540 -0.782838 0.973576

C 3.568871 0.617711 -0.748728

C 3.764327 -1.556231 0.973931

C 4.712190 -0.165292 -0.746665

C 4.810971 -1.253750 0.111857

H 1.807299 -1.008287 1.656478

H 3.495120 1.461603 -1.422095

H 3.843477 -2.394753 1.652350

H 5.526460 0.072445 -1.417837

H 5.704378 -1.864078 0.109917

(**5**) Int_exo_Ph

28

C -3.067309 -2.703613 -0.106910

C -1.951044 -1.901969 -0.130239

C -4.389414 -2.197815 0.024123

H -0.962927 -2.326459 -0.232023

H -5.222402 -2.886868 0.038673

C -2.142676 -0.512327 -0.012391

C -4.614368 -0.855711 0.133849

H -5.595817 -0.414935 0.238757

C -3.463272 -0.057458 0.102678

H -2.938946 -3.774037 -0.191544

N -3.424566 1.304802 0.205308

O -2.172891 1.744221 0.149233

O -4.314819 2.093607 0.338071

C -1.304939 0.644852 0.015541

C 0.047313 0.836902 0.009812

Au 1.229129 -0.786864 0.024865

Cl 2.556637 -2.760512 0.026756

C 0.623184 2.182414 -0.004569

C 1.821107 2.411388 0.690535

C 0.058336 3.242223 -0.732383

C 2.405061 3.665641 0.700578

C 0.670639 4.483873 -0.754499

C 1.836015 4.703195 -0.029023

H 2.279753 1.592536 1.229775

H -0.836719 3.081095 -1.315858

H 3.317893 3.828122 1.257700

H 0.239474 5.283701 -1.341582

H 2.307772 5.676486 -0.044350

(**6**) RC (R = -CH_2_CH_3_)

24

C -2.954864 2.474967 0.201051

C -1.951697 1.524452 0.114350

C -2.646421 3.827082 0.196364

H -2.205204 0.473664 0.119818

H -3.425124 4.573430 0.264262

C -0.606565 1.890479 0.015966

C -1.324848 4.223868 0.100398

H -1.047905 5.267079 0.091690

C -0.327195 3.268363 0.011171

H -3.983127 2.150064 0.271754

N 1.045875 3.781760 -0.092660

O 1.956871 2.991454 -0.166388

O 1.195611 4.980758 -0.098961

C 0.406766 0.866846 -0.076410

C 1.458654 0.236800 -0.177400

Au -0.125575 -1.295224 -0.036762

Cl -1.326523 -3.290430 0.057560

C 2.866891 -0.155073 -0.293401

H 3.003636 -0.718092 -1.218087

H 3.427726 0.777460 -0.388904

C 3.367908 -0.964922 0.903751

H 2.846590 -1.919030 0.975376

H 3.217479 -0.416203 1.832192

H 4.432757 -1.163595 0.789004

(**7**) TS_endo_Et

24

C -4.128706 -0.793924 -0.596366

C -2.766351 -0.893054 -0.808648

C -4.668162 0.315048 0.051330

H -2.346473 -1.752454 -1.313471

H -5.732871 0.390730 0.218029

C -1.892201 0.108934 -0.381663

C -3.836387 1.325802 0.485876

H -4.224570 2.197123 0.990995

C -2.466467 1.212717 0.266910

H -4.777757 -1.587635 -0.938576

N -1.655811 2.310630 0.753668

O -0.429236 2.300416 0.594244

O -2.190167 3.229500 1.309501

C -0.465159 -0.006232 -0.614163

C 0.459822 0.807930 -0.318693

Au 0.498441 -1.640161 -1.506270

Cl 1.470496 -3.532943 -2.515699

C 1.839229 1.287041 -0.215550

H 2.300425 1.124093 -1.191981

H 1.822355 2.364650 -0.050408

C 2.636714 0.577105 0.882150

H 2.664307 -0.496321 0.696961

H 2.194469 0.753353 1.861640

H 3.659211 0.952378 0.889434

(**8**) TS_exo_Et

24

C -4.066805 -0.676155 0.113325

C -2.705044 -0.833440 -0.087867

C -4.648772 0.573469 0.356571

H -2.277183 -1.808429 -0.275808

H -5.715346 0.654250 0.508404

C -1.886978 0.297925 -0.043879

C -3.860651 1.705264 0.403012

H -4.255459 2.695227 0.586479

C -2.504864 1.519019 0.199500

H -4.699290 -1.552256 0.079757

N -1.567370 2.592938 0.222548

O -0.390980 2.243855 0.013605

O -1.888281 3.725360 0.426558

C -0.462773 0.361899 -0.225271

C 0.686358 -0.139772 -0.447239

Au 0.434835 -2.193361 -0.693472

Cl 0.266784 -4.527520 -1.011041

C 2.054153 0.447098 -0.559691

H 2.427951 0.219390 -1.559872

H 1.993458 1.531009 -0.461562

C 3.016084 -0.133221 0.478508

H 3.120004 -1.211396 0.354856

H 2.661510 0.062071 1.490166

H 3.998520 0.323474 0.363967

(**9**) Int_endo_Et

24

C 2.992556 2.409862 -0.205096

C 2.072598 1.403796 -0.105986

C 2.605174 3.774253 -0.217662

H 2.372229 0.364985 -0.096638

H 3.356817 4.547358 -0.295752

C 0.690705 1.691585 -0.003319

C 1.290865 4.113106 -0.128912

H 0.949736 5.137667 -0.133692

C 0.348942 3.064380 -0.019344

H 4.043524 2.164999 -0.272918

N -0.970811 3.413950 0.070162

O -1.894059 2.473785 0.214387

O -1.398505 4.537180 0.039897

C -0.308038 0.681631 0.123428

C -1.583236 1.137091 0.248921

Au 0.081377 -1.305267 0.087607

Cl 0.533324 -3.641013 0.027514

C -2.843449 0.349819 0.388885

H -2.625297 -0.509665 1.021739

H -3.593006 0.963389 0.889959

C -3.367199 -0.123549 -0.971125

H -2.633721 -0.767576 -1.456612

H -3.580802 0.722100 -1.625203

H -4.286661 -0.690836 -0.833402

(**10**) Int_exo_Et

24

C -2.852686 2.396088 0.209728

C -1.825303 1.485630 0.125453

C -2.651545 3.802757 0.195525

H -2.015155 0.421759 0.137022

H -3.506713 4.460103 0.262842

C -0.518133 1.992122 0.019852

C -1.394307 4.328635 0.096141

H -1.180404 5.388240 0.081292

C -0.365038 3.383469 0.011721

H -3.866684 2.028170 0.289682

N 0.974768 3.652042 -0.100377

O 1.676527 2.531439 -0.163612

O 1.542730 4.702199 -0.145075

C 0.791640 1.431309 -0.094952

C 1.288425 0.169770 -0.143090

Au 0.036164 -1.385882 -0.051333

Cl -1.492239 -3.211043 0.049446

C 2.763054 -0.052880 -0.266837

H 2.914831 -0.592589 -1.206855

H 3.327486 0.877989 -0.324590

C 3.292980 -0.921514 0.879728

H 2.779084 -1.881723 0.902959

H 3.139232 -0.431286 1.841005

H 4.359959 -1.099424 0.751826

(**11**) D

28

C 3.074321 2.487515 -0.232907

C 2.308479 1.450597 0.176774

C 2.529614 3.748555 -0.682713

H 2.742211 0.527099 0.524318

H 3.203449 4.533344 -0.995915

C 0.867195 1.593435 0.186594

C 1.196910 3.942517 -0.707173

H 0.735643 4.865212 -1.030264

C 0.364911 2.865380 -0.267949

H 4.150618 2.375199 -0.223055

N -0.957975 2.928279 -0.225438

O 1.264726 -0.982596 1.514078

O -1.660610 3.858527 -0.588982

C -0.038190 0.641795 0.560534

C 0.201636 -0.742003 0.973836

Au -1.955146 1.340072 0.556486

Cl -3.554628 -0.036066 1.528932

C -0.789638 -1.815830 0.721318

C -1.529628 -1.869808 -0.455367

C -0.924106 -2.811535 1.685420

C -2.413130 -2.917918 -0.663177

C -1.826042 -3.842593 1.486085

C -2.569301 -3.894980 0.311973

H -1.405954 -1.102383 -1.211225

H -0.328752 -2.755060 2.587435

H -2.985817 -2.967899 -1.579109

H -1.949472 -4.606105 2.242569

H -3.270887 -4.703513 0.155935

(**12**) E

28

C 3.054470 2.556536 -0.042995

C 2.285203 1.450920 0.029618

C 2.498225 3.872354 -0.223725

H 2.737905 0.482356 0.154644

H 3.155340 4.729091 -0.272187

C 0.835039 1.510239 -0.066073

C 1.170389 4.015819 -0.337180

H 0.701601 4.979713 -0.478374

C 0.278893 2.871367 -0.252368

H 4.129205 2.451516 0.031700

N -0.978639 3.186421 -0.337165

O 1.665621 -1.287769 0.697624

O -1.963677 2.381002 -0.228805

C 0.092819 0.376389 0.002393

C 0.533122 -0.963526 0.413033

Au -1.898124 0.351986 -0.162081

Cl -4.374629 0.231220 -0.304407

C -0.593197 -1.938090 0.556260

C -1.590202 -2.004125 -0.443111

C -0.659880 -2.757856 1.678751

C -2.673858 -2.888431 -0.273215

C -1.720069 -3.635607 1.810523

C -2.733831 -3.691322 0.844379

H -1.369562 -1.650543 -1.450296

H 0.118929 -2.702194 2.428193

H -3.419291 -2.970799 -1.052219

H -1.766401 -4.289480 2.671820

H -3.546121 -4.395063 0.964425

(**13**) F

28

C -2.752204 2.649171 -0.387430

C -1.610714 1.864503 -0.487529

C -2.677423 3.961044 0.072820

H -1.674884 0.825530 -0.777266

H -3.571511 4.561889 0.165069

C -0.360011 2.379433 -0.166548

C -1.448612 4.498395 0.403274

H -1.350508 5.518454 0.747739

C -0.307895 3.710540 0.249803

H -3.713474 2.229177 -0.652561

N 0.965823 4.298928 0.498136

O 0.457086 0.199548 -0.043843

O 1.004746 5.268166 1.187592

C 0.784650 1.385919 -0.062386

C 2.118718 1.711693 0.325286

Au 2.756787 3.698177 -0.442447

Cl 4.721913 3.634251 -1.689713

C 3.222773 0.889364 -0.095079

C 4.344443 0.793761 0.746464

C 3.225047 0.194127 -1.320303

C 5.434335 0.027558 0.380494

C 4.323255 -0.551107 -1.693929

C 5.426769 -0.632237 -0.844629

H 4.327901 1.330980 1.685711

H 2.361081 0.265354 -1.967996

H 6.291985 -0.050595 1.034568

H 4.333587 -1.066547 -2.644345

H 6.287533 -1.216780 -1.143018

(**14**) G

24

C 3.123734 2.542657 -0.234724

C 2.369792 1.419792 -0.192303

C 2.577364 3.877505 -0.144159

H 2.810239 0.441640 -0.275377

H 3.243863 4.728205 -0.179231

C 0.930248 1.530812 -0.051886

C 1.249543 4.054633 -0.016857

H 0.780330 5.025255 0.057565

C 0.430681 2.881664 0.023055

H 4.195870 2.442951 -0.342032

N -0.884013 2.925229 0.141184

O 1.419576 -1.288979 -0.221614

O -1.591196 3.911903 0.261019

C 0.023041 0.506318 0.018234

C 0.292832 -0.939060 0.073935

Au -1.911803 1.185754 0.156307

Cl -3.768204 -0.227398 0.177791

C -0.730421 -1.938838 0.528561

H -1.664275 -1.800572 -0.013095

H -0.323169 -2.916686 0.275025

C -0.974391 -1.841550 2.036219

H -1.351649 -0.859171 2.324878

H -0.053183 -2.031196 2.588394

H -1.714964 -2.580700 2.336381

(**15**) H

24

C 2.940401 2.240362 -0.051789

C 1.933607 1.444575 0.332892

C 2.709891 3.553679 -0.614631

H 2.140775 0.479310 0.770132

H 3.555002 4.158151 -0.911256

C 0.526875 1.858698 0.221090

C 1.460397 4.007473 -0.760195

H 1.238108 4.979541 -1.177692

C 0.312616 3.206536 -0.365634

H 3.958368 1.892953 0.066232

N -0.839945 3.751280 -0.575071

O 0.405486 -0.424544 2.297792

O -1.956405 3.183690 -0.292828

C -0.408667 1.004447 0.641081

C -0.170816 -0.368025 1.245507

Au -2.345897 1.479734 0.694905

Cl -3.331807 -0.285505 1.850721

C -0.563671 -1.583851 0.453279

H -1.533126 -1.415905 -0.014651

H 0.166487 -1.650659 -0.362699

C -0.556401 -2.840041 1.309101

H -1.274800 -2.743228 2.123874

H 0.426737 -3.006866 1.746815

H -0.824533 -3.708753 0.710440

(**16**) I

24

C -2.654774 2.546707 -0.206243

C -1.506442 1.806055 -0.434121

C -2.580173 3.855400 0.272714

H -1.560433 0.782673 -0.775103

H -3.480877 4.425044 0.455170

C -0.242843 2.348847 -0.209174

C -1.347650 4.419561 0.510246

H -1.251524 5.432613 0.873683

C -0.194138 3.662000 0.266734

H -3.622435 2.101012 -0.396106

N 1.064527 4.254871 0.519667

O 0.620904 0.210602 -0.607965

O 1.089453 5.252960 1.175561

C 0.916508 1.386764 -0.359025

C 2.295038 1.678883 -0.375166

Au 2.966392 3.699275 -0.212909

Cl 5.240924 3.618903 -0.724334

C 3.226526 0.855183 0.435229

H 2.931839 -0.160275 0.135013

H 4.262063 1.006063 0.136409

C 3.060967 0.982114 1.955058

H 2.025456 0.797465 2.245803

H 3.347390 1.978377 2.297968

H 3.697434 0.259301 2.464860
